# Heartache in a Bottle: Understanding Alcoholic Cardiomyopathy

**DOI:** 10.7759/cureus.42886

**Published:** 2023-08-03

**Authors:** Nkechinyere L Ihekire, Okelue E Okobi, Enoobong Aderonke Adedoye, Patience F Akahara, Anita O Onyekwere, Jeffrey Afrifa-Yamoah, Falilatu B Akinyemi

**Affiliations:** 1 Internal Medicine, Windsor University School of Medicine, Cayon, KNA; 2 Family Medicine, Medficient Health Systems, Laurel, USA; 3 Family Medicine, Lakeside Medical Center, Belle Glade, USA; 4 Internal Medicine, Mater Dei Hospital, Msida, MLT; 5 Family Medicine, Inglewood Medical Centre, Edmonton, CAN; 6 School of Medicine, Richmond Gabriel University, Kingstown, VCT; 7 Internal Medicine, Korle Bu Teaching Hospital, Accra, GHA

**Keywords:** alcoholic heart disease, alcohol-induced cardiomyopathy, alcoholic cardiomyopathy (acm), dilated cardiomyopathy (dcm), alcohol

## Abstract

Alcoholic cardiomyopathy (ACM) is a cardiac ailment marked by impaired contraction and dilation of one or both ventricles of the heart. The extent of daily alcohol intake and duration of alcohol abuse are linked to the development of ACM, although the exact thresholds and timeline for alcohol misuse to induce heart dysfunction remain uncertain. Thus, the objective of this systematic review is to comprehensively evaluate the existing knowledge on the specific disease entity, particularly in light of the ongoing issue of alcohol misuse, with the intention of determining if recent advancements and discoveries have significantly altered the understanding of this condition compared to the past century. This systematic review involved a literature search that was conducted on PubMed to identify suitable and appropriate literature for the study. The inclusion criteria encompassed articles that focused on ACM or the relationship between alcohol abuse and cardiac dysfunction, involved human subjects or relevant animal models, were written in the English language, and were published within the last 10 years. The exclusion criteria included duplicates, case reports, letters, editorials, and reviews not specifically addressing ACM. As a result, a total of 18 articles were included in this systematic review. The risk of bias was assessed through the use of the Cochrane risk-of-bias tool for clinical trials. The findings of this systematic review indicated that the likelihood of ACM occurrence significantly rose when the consumption of over 80 g of alcohol per day occurred for at least five years. The systematic review further revealed that ACM is associated with various detrimental changes in the cellular, structural, and histological aspects of the heart muscles, even though the specific clinical and histological characteristics of ACM have yet to be established. In individuals with an extensive history of excessive alcohol abuse, the diagnosis of ACM was reached through the exclusion of other potential causes of the condition. The fundamental approach to treatment lies in abstaining from alcohol. It is crucial to manage symptoms in individuals with secondary heart failure and address any related complications.

## Introduction and background

Alcoholic cardiomyopathy (ACM), initially identified in 1877, is a cardiac condition where the contraction and dilation of one or both ventricles of the heart are impaired, despite normal or reduced ventricular wall thickness. It is associated with a prolonged history of alcohol abuse and has no other known causes. Histological examination of tissue samples often reveals signs of dilatation, myofibrillar necrosis, fibrosis, reduced myofibrils, and enlarged mitochondria. However, these findings lack specificity and do not differentiate ACM from other forms of dilated cardiomyopathy (DCM) [1].

Based on epidemiological evidence, ACM is recognized as a significant contributor to non-ischemic DCM in Western countries. Diagnosing ACM still relies on exclusion criteria, similar to alcoholic liver disease, as excessive alcohol consumption is observed in up to 40% of DCM patients. In a national inpatient sample study, some authors have reported ACM to be most common in white males aged between 45 and 59 [2].

The quantity of alcohol consumed daily and the duration of alcohol misuse are linked to the development of ACM, although the precise thresholds for causing cardiac dysfunction remain unknown. The risk of ACM significantly increases with alcohol intake exceeding 80 g per day for a minimum of five years [3]. According to the American Heart Association (AHA) and other US-based guidelines, alcohol intake recommendations are provided to promote responsible drinking habits and maintain overall health. The AHA suggests moderate alcohol consumption for those who choose to drink, defining moderation as up to one drink per day for women and up to two drinks per day for men. It is important to note that these guidelines apply to healthy adults and should be adjusted for individuals with certain health conditions or those taking specific medications. In addition, the AHA advises against starting to drink alcohol solely for potential health benefits, as the risks can outweigh the advantages. It is crucial to exercise caution and be aware of individual tolerance and personal health circumstances when making decisions about alcohol consumption. Consulting with a healthcare professional can provide personalized advice and guidance. Furthermore, they specified the definition of “one drink” offer clarity when it comes to alcohol consumption. The guidelines typically define one drink as specific quantities for different types of alcoholic beverages. For instance, a single drink of beer is typically considered as a 12-ounce (355 ml) serving of regular beer, usually containing around 5% alcohol by volume (ABV). When it comes to wine, one drink is defined as a 5-ounce (148 ml) serving, which typically contains about 12% ABV. Distilled spirits, such as vodka, whiskey, rum, or tequila, are measured as 1.5 ounces (44 ml) per drink, with a typical ABV of around 40%. It is important to note that the size and strength of different alcoholic beverages can vary, so these definitions serve as general guidelines. It is always advisable to be mindful of individual tolerance and consume alcohol responsibly [4-6].

The cumulative amount of ethanol consumed throughout a person's lifetime, expressed as kilograms per kilogram of body weight (kg/kg), is referred to as the "total lifetime dose of ethanol" (TLDE), and it appears to be associated with left ventricular dysfunction. However, not all individuals classified as "heavy drinkers" develop DCM. The most effective approach for managing alcohol-related organ damage, including ACM, and promoting recovery of left ventricular dysfunction, is complete abstinence from alcohol. The exact "point of no return" or the threshold between reversible and irreversible damages is yet to be fully understood [1-3]. The objective of this scholarly paper is to comprehensively examine existing knowledge on the specific disease entity, particularly in light of the ongoing issue of alcohol misuse, with the intention of determining if recent advancements and discoveries have significantly altered our understanding of this condition compared to the past century.

Review methodology

In this literature review on the topic "heartache in a bottle: understanding alcoholic cardiomyopathy," we employed a methodology that involved utilizing the PubMed database as our primary repository for collecting relevant scholarly articles. We chose PubMed due to its comprehensive coverage of biomedical literature, including studies related to cardiovascular diseases and alcohol-induced cardiomyopathy. To ensure a systematic approach, we followed several steps to identify and select appropriate articles for inclusion in this review.

First, we devised a search strategy to retrieve relevant articles from PubMed. We used search terms, such as "alcoholic cardiomyopathy," "alcohol-induced cardiomyopathy," "alcohol-related heart disease," "alcohol abuse and heart failure," and "alcohol and cardiac dysfunction." By combining these terms using Boolean operators, we aimed to enhance the search precision and coverage. Next, we established inclusion and exclusion criteria to determine the eligibility of articles. Inclusion criteria encompassed articles that focused on ACM or the relationship between alcohol abuse and cardiac dysfunction, involved human subjects or relevant animal models, were written in the English language, and were published within the last 10 years. Meanwhile, we excluded duplicates, case reports, letters, editorials, and reviews not specifically addressing ACM. We then proceeded with screening and selection based on the titles and abstracts of the initial search results. Two independent reviewers assessed each article for relevance and eligibility for full-text review. In cases of disagreement, we reached a consensus through discussion. Full-text articles were obtained for the selected studies. Once the 15 articles were selected (see Appendix Table 1 for the list of included articles), we extracted and organized relevant information from them. This information included study objectives, design, sample size, patient characteristics, experimental procedures, outcome measures, and main findings, including the existing association between increased daily alcohol intake and development of ACM and ACM's association with harmful cellular, structural, and histological alterations in the myocardium that affect the heart's systolic and diastolic function.

The effect measure for each outcome was conducted using the mean differences effect measure, where the outcomes were assessed in identical units across the various literature reviews used in the study. Furthermore, for this review, certainty assessment was conducted by assessing the risk of bias, imprecision, inconsistency, and indirectness of the presented evidence. Through a thematic synthesis, we identified common trends, knowledge gaps, and emerging research areas related to ACM. To assess the quality and validity of the included studies, we performed a critical appraisal using appropriate tools such as the Newcastle-Ottawa Scale for observational studies or the Cochrane Risk of Bias tool for clinical trials. This assessment allowed us to evaluate the methodological rigor of each study and determine its overall quality and potential impact on the literature review. Finally, we analyzed and presented the synthesized literature, along with relevant findings and conclusions from the included studies, in a coherent manner. We identified main themes and sub-themes to provide a comprehensive overview of the current state of knowledge regarding ACM. By following this methodology, we aim to contribute to the existing body of knowledge on ACM, providing a reliable and up-to-date understanding of its pathogenesis, clinical features, diagnostic approaches, treatment options, and potential preventive strategies.

## Review

Genetics

The emergence of next-generation genome sequencing has facilitated the identification of genetic variations within sarcomeric protein-encoding genes, which can be linked to different types of cardiomyopathies, including hypertrophic remodeling and ventricular dilatation. These proteins play a crucial role in generating contractile force and maintaining cellular anchoring. Mutations in sarcomeric protein-coding genes associated with DCM can potentially hinder the body's ability to adapt to physiological stress. Of particular concern in DCM is the sarcomeric protein called titin, encoded by the TTN gene. Different variants of TTN can result in protein truncation. Approximately 25% of familial DCM cases, which are inherited in an autosomal dominant manner, and 12-18% of sporadic DCM cases may be attributed to TTN variations. However, variations in TTN are typically not associated with skeletal muscle dysfunction or cardiac conduction abnormalities. Titin cardiomyopathy, which typically manifests in the fifth to sixth decades of life, presents similarly to idiopathic forms of DCM. The prognosis is comparable, but individuals with titin cardiomyopathy may respond more favorably to medical interventions compared to those with idiopathic DCM [7-9].

Pathophysiology

The pathophysiology of ACM describes how the ACM's biological and physical symptoms are linked to the underlying distortion and physiological imbalances. Long-term alcohol consumption is directly associated with ACM. Alcoholism is still thought to be the leading cause or contributory factor in secondary non-ischemic DCM, accounting for up to one-third of all cases with DCM. Numerous research studies have documented that ACM is linked to a diverse range of detrimental changes at the cellular, structural, and histological levels within the myocardium. Ventricle enlargement can occur as a result of original cardiomyopathy or subsequent left ventricular failure. Moreover, there is an effect on both the systolic and diastolic functions due to the increase in end-diastolic and end-systolic volumes caused by cardiac remodeling. Cardiac remodeling, which is a process where the heart undergoes structural changes in response to certain conditions (e.g., chronic hypertension or heart failure), can have an impact on both the systolic and diastolic functions of the heart. The increase in end-diastolic volume (the volume of blood in the ventricle at the end of diastole) and end-systolic volume (the volume of blood in the ventricle at the end of systole) can affect the heart's ability to contract and relax during each cardiac cycle. This, in turn, can have implications for overall heart function [1,7-9]. These continuous ventricular dilatations may lead to other structural distortions, such as mitral and tricuspid valve regurgitation yielding an overall reduction of the ejection fraction. The primary pathogenic components of ACM include the induction of oxidative-stress process, increased protein catabolism, alteration of lipid transport and metabolism, apoptosis of myocytes, and disturbed mitochondrial biogenetics [9].

Oxidative stress

According to current knowledge, prolonged and excessive alcohol consumption plays a significant role in inducing oxidative stress within the myocardium. This can occur through direct means, by promoting the generation of free radicals, or indirectly, by triggering the release of hormones, such as angiotensin II, or activating other systems. Moreover, alcohol may reduce the levels of transport proteins and diminish antioxidant activity by decreasing the plasma concentration of antioxidant enzymes. These mechanisms contribute to the development of oxidative stress, which is responsible for the onset of cardiomyopathies and ischemia-reperfusion injury. The aforementioned processes can have various adverse effects on the heart, including distortion and loss of myocardial cells, alterations in the sarcoplasmic reticulum, abnormalities in intracellular calcium regulation, mitochondrial dysfunction, reduced activity of myofibrillar ATPase and calcium sensitivity, disruption of contractile proteins, and accumulation of fatty acids within intracellular organelles [10].

Prior studies have investigated the impact of ethanol on changes in the activity and levels of oxidative enzymes. Catalase activity is significantly increased in postmortem heart samples acquired from people who have been diagnosed with ACM. Other studies investigated the catalase levels and activity among rats with ACM with a control group. They demonstrated a much higher catalase activity among rats suffering from ACM. This may be explained by the fact that the increased catalase activity in those who have a long history of alcohol abuse may represent a protective and adoptive reaction to the persistent high ethanol levels [11].

Apoptotic cell death

Apoptosis occurs mainly as a consequence of lipid peroxidation and oxidative stress in various body organs. There is a significant association between cardiovascular disorders and apoptosis. There is also an established link between the development of ACM and apoptosis because of myocardial cell death, which contributes to heart pathology and dysfunction. Previous studies were conducted on rats that are fed alcohol for about eight months. They found that there is about 14% loss of myocardial cells in the left ventricle of those rats. Caspase-3 is one of the most reliable apoptotic markers in the body. It showed a significant increase in both acute and chronic alcohol intoxication. The exact mechanism of apoptosis is still not clear, and many factors may be involved in this process, including the end products of alcohol metabolism, such as acetaldehyde; the release of angiotensin II that contribute to myocyte damage, cell death, remodeling, and cardiomyopathy in a protein kinase C/nicotinamide adenine dinucleotide phosphate oxidase-dependent manner; and released growth factors, such as insulin-like growth factor I (IGF-I) and myostatin. All previous mechanisms can induce myocyte apoptosis through the induction of mitochondrial damage and oxidative stress [12].

Impaired mitochondrial bioenergetics/stress

Evidence of altered bioenergetics or mitochondrial dysfunction has been observed in various investigations of ethanol effect on the heart. Disrupted bioenergetics and oxidative phosphorylation indices and a change in the ultrastructure of the mitochondria may be the cause of such dysfunctions. This can be understood through clinical observations that highlight the mitochondria as the main target of oxidative damage. When reactive oxygen species (ROS) are produced in excessive manners due to heavy alcohol consumption, it damages mitochondrial DNA, resulting in mitochondrial injuries. Surprisingly, the damaged mitochondria not only become less efficient but also increases the generation of ROS that aid the apoptosis process. Furthermore, in contrast to nuclear DNA, mitochondrial DNA is susceptible to oxidative stress due to its close proximity to the formation of ROS and the limited protective mechanisms in place to safeguard DNA integrity. Post-mortem biopsies from the hearts of human alcoholics revealed that the myocardial mitochondria is enlarged and damaged [1-9].

Abnormalities in fatty acid metabolism and transport

Excessive alcohol consumption may alter fatty acid uptake and metabolism. The formation of fatty ethyl esters (FAEEs) has been implicated in ethanol-induced cell injury. ACM can occur as a result of an increase in FAEE concentration and probable changes in the FAEE synthase enzyme. Thus, alcohol consumption increases the uptake of long-chain fatty acid, and triglyceride accumulation of which is significantly associated with the reduction in myocardial adenosine triphosphate (ATP) levels and myocardial contractility [7]. As a result of the aforementioned intracellular alterations, the human body triggers compensatory mechanisms, including the release of natriuretic peptides and cytokines, hyperactivation of the sympathetic nervous system, and activation of the renin-angiotensin-aldosterone system. These mechanisms play a role in the pathophysiology of ACM, characterized by left ventricular dilation, decreased cardiac output, elevated preload, and hypertrophy of unaffected myocytes [2-4].

Clinical manifestations

The clinical features of ACM develop when the injury is irreversible and advanced. Therefore, based on the existence or absence of congestive heart failure symptoms and signs, individuals may be classified as asymptomatic (preclinical phase) or symptomatic (clinical phase). Left or both ventricles may enlarge and exhibit poor contraction in ACM. The left ventricular end-diastolic diameters show a significant increase in such patients compared to healthy individuals in the same age and weight. Moreover, there is a decrease in the left ventricular mass index and ejection fraction, falling below the normal range. Diastolic dysfunction, characterized by impaired left ventricular relaxation and reduced diastolic filling capacity, serves as an early indicator of ACM. Ventricular dilatation is the first echocardiographic change seen in alcohol use disorder patients, coming before diastolic dysfunction and hypertrophy. The symptoms of left ventricular diastolic function included waking up at night with shortness of breath, irregular heartbeat, extreme fatigue and weakness, dizziness and fainting, bouts of chest pain, and swelling in the feet, ankles, and abdomen [13].

Diagnosis

**Figure 1:**
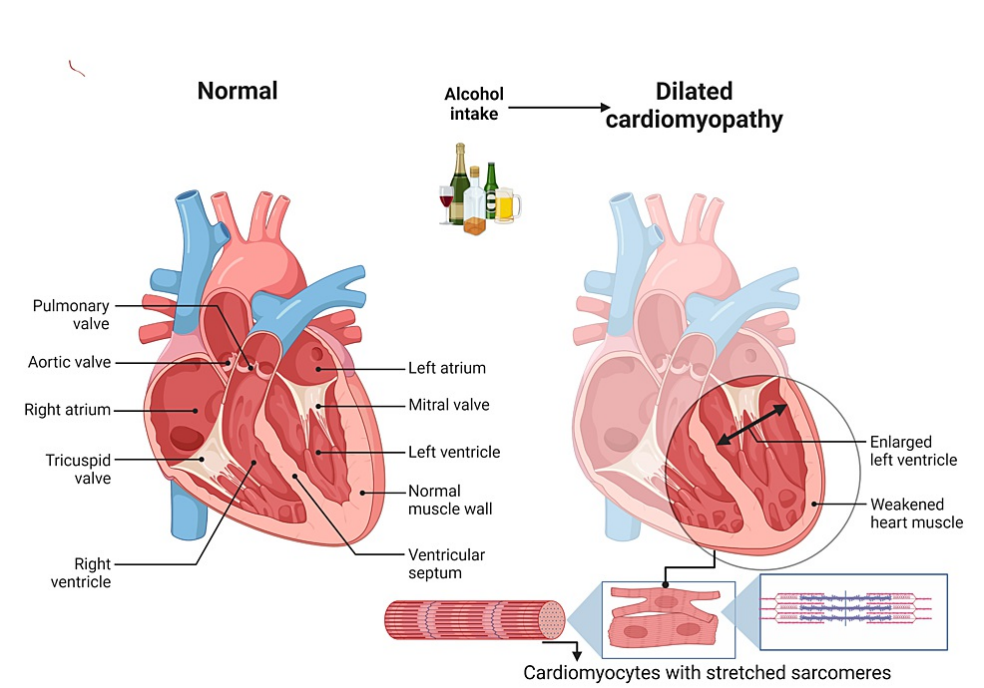
Alcohol-induced dilated cardiomyopathy This image was an original creation of the authors of this manuscript and has been created using EdrawMax 11.5.0 (Wondershare, China) for Microsoft Windows.

Both specific clinical and histological characteristics of ACM have not yet been determined. In individuals with a prolonged history of alcohol misuse, the diagnosis of ACM is established through the exclusion of other potential causes of DCM. It is particularly important to consider excessive alcohol consumption in individuals who have cultural, social, or health conditions that place them at risk for alcohol intake. The Diagnostic and Statistical Manual of Mental Disorders, Fifth Edition (DSM-5) criteria, along with guidance from a healthcare professional specializing in alcohol addiction, can assist in the diagnostic process. Blood tests, such as mean corpuscular volume (MCV), ethyl-glucuronide, carbohydrate-deficient transferrin (CDT), and gamma glutamyl-transpeptidase (GGT), can be utilized to confirm suspicions of alcohol addiction, rule out co-existing alcoholic liver disease, and monitor alcohol abstinence. Pleural effusion, pulmonary congestion, and cardiomegaly can all be shown on a chest X-ray. The ECG is typically non-specific because it can reveal changes in ST segments, T-waves, and any arrhythmias that result from dilated heart chambers. In addition to identifying characteristics associated with alcoholic cardiomyopathies, such as hypertrophy, dilatation (see Figure 1), and systolic and diastolic dysfunction, echocardiography also allows for the exclusion of other potential causes of heart failure. Ultimately, the diagnosis of ACM primarily relies on the association between long-term alcohol abuse and myocardial abnormalities that cannot be attributed to other evident cardiac conditions. In addition, a significant aspect of ACM diagnosis is the improvement in heart function observed after discontinuation of alcohol consumption [14,15].

Management

The goal of treatment is source control. Alcohol abstinence is the cornerstone of treatment. Counseling and resource provision for patients should be part of management. Symptomatic management in people with secondary heart failure to address any related consequences is also vital in managing ACM. According to several articles, even moderate alcohol use has comparable effects to abstinence. Goal-directed heart failure therapy, as utilized in idiopathic DCM with low ejection fraction, should be a part of pharmaceutical therapy. If the left ventricular ejection fraction (LVEF) is less than or equal to 40%, this may also comprise a combination of angiotensin blocker-neprilysin inhibitor, diuretics, beta-blockers, diuretics, aldosterone receptor antagonists, and an angiotensin-converting enzyme inhibitor. Some studies have shown that the combination of carvedilol and trimetazidine with other traditional heart failure medications is effective [1-3,7-11,16-20].

Prognostic indicators

The long-term survival rate is directly influenced by the extent of alcohol consumption and the duration of its use. Alcohol-related DCM exhibits a more favorable outlook compared to cardiomyopathy caused by ischemia. Unfavorable outcomes are associated with atrial fibrillation, QRS widening exceeding 120 ms, and the absence of beta-blockers. Patients who persist in consuming alcohol exhibit a bleak prognosis. Evidence suggests that individuals who successfully abstain from alcohol experience improved overall outcomes, including a decrease in hospital admissions and a reduction in the size of the heart as observed on an echocardiogram. Continued alcohol consumption may lead to escalating heart failure, arrhythmias, and cardioembolic events. Data indicates that depending on the type of alcohol consumed, mortality rates of 40-80% are observed within a decade [2,17-18].

## Conclusions

ACM is a major cause of non-ischemic DCM. There is a significant association between the amount of alcohol consumed daily and the development of the condition. The main pathological components of ACM include the induction of oxidative stress process, increased protein catabolism, alteration of lipid transport and metabolism, apoptosis of myocytes, and disturbed mitochondrial biogenetics. ACM is associated with a variety of harmful cellular, structural, and histological alterations in the myocardium that affect both systolic and diastolic functions. The cornerstone of treating ACM is alcohol abstinence.

## Appendices

**Table 1:** List of literature articles reviewed in this study List of the 15 articles reviewed in this study, indicating the study authors, objectives, design, sample size, patient characteristics, experimental procedures, outcome measures, and main findings.

Study	Study objectives	Design	Sample size	Patient characteristics	Experimental procedures	Outcome measures	Main findings
Fernández-Solà J: *The effects of ethanol on the heart: Alcoholic cardiomyopathy *[1]	To describe and discuss the global effects that ethanol exerts on the heart myocytes, the so-called alcoholic cardiomyopathy (ACM).	Literature review	135 studies	N/A	N/A	N/A	Control of other cardiac risk factors also allows for a better prognosis in ACM. Total abstention from alcohol is the preferred goal, although controlled drinking (with daily consumption <60 g/day) still allows improvement. Binge drinking should be absolutely discouraged in ACM. Subjects with ACM who continue in high-dose ethanol consumption have a bad prognosis, with repeated episodes of heart failure and ventricular arrhythmias leading to a 10% increase in annual mortality rate.
Ram P, Lo KB, Shah M, Patel B, Rangaswami J, Figueredo VM: *National trends in hospitalizations and outcomes in patients with alcoholic cardiomyopathy *[2]	To determine the trends in hospitalization among patients with AC and associated outcomes for better understanding of the disease.	Hospital discharge data analysis	45,365 patients	Only patients with AC were included in the study.	N/A	N/A	Although the overall admissions among patients with AC decreased over time, the proportion of patients with high-risk characteristics, such as smoking, depression, and drug abuse, increased. Patients aged 45 and older were largely affected, and cardiovascular etiologies predominated among causes for admission.
Addolorato G, Mirijello A, Barrio P, Gual A: *Treatment of alcohol use disorders in patients with alcoholic liver disease *[3]	To summarize current treatments for alcohol use disorder (AUD) with a particular emphasis on treatment of AUD in patients with ALD and to analzse medical management, psychosocial and pharmacological interventions, underlying limits, and options in AUD patients. To discuss the most appropriate setting for the management of AUD patients with advanced liver disease and the indications for liver transplantation in AUD patients.	Narrative review	62 studies	N/A	N/A	N/A	AUDs represent the most common cause of ALD in the Western world. The achievement and maintenance of total alcohol abstinence remains the cornerstone of treatment. The combination of psychosocial interventions, pharmacological therapy, and medical management seems to be the most effective management strategy for AUD patients with ALD.
Mirijello A, Tarli C, Vassallo GA, et al.: *Alcoholic cardiomyopathy: What is known and what is not known* [7]	To describe the clinical features of alcoholic cardiomyopathy, particularly emphasizing on the areas of uncertainty of this under-recognized disease.	Narrative review	53 studies	N/A	N/A	N/A	Although alcoholic cardiomyopathy is a well-known disease, there are still several pitfalls in the pathophysiology, natural history, diagnosis, and risk stratification of this disease.
Rosenbaum AN, Agre KE, Pereira NL: *Genetics of dilated cardiomyopathy: practical implications for heart failure management* [8]	To improve the understanding of the cardiomyopathy disease processes to facilitate better clinical decision-making about the therapies offered, exemplifying the implementation of precision medicine.	Literature review	142 studies	N/A	N/A	N/A	Focused evaluation of the clinical characteristics and inheritance pattern of heart failure or sudden cardiac death, in addition to consultation with a genetic counsellor when appropriate, can facilitate a successful genetic evaluation. Moreover, genetic testing is recommended in all patients with familial dilated cardiomyopathy (DCM) to facilitate screening, whereas guideline recommendations for testing in patients with sporadic DCM differ, but specific clinical features might increase the yield of testing.
Piano MR, Phillips SA: *Alcoholic cardiomyopathy: pathophysiologic insights *[9]	To provide a mechanistic paradigm for future research in the area of ACM.	Literature review	97 studies	N/A	N/A	N/A	Several mechanisms are implicated in mediating the adverse effects of ethanol, including the generation of oxidative stress, apoptotic cell death, and impaired mitochondrial bioenergetics/stress, derangements in fatty acid metabolism and transport, and accelerated protein catabolism.
Matyas C, Varga ZV, Mukhopadhyay P, et al.: *Chronic plus binge ethanol feeding induces myocardial oxidative stress, mitochondrial and cardiovascular dysfunction, and steatosis *[10]	To describe mouse models of alcoholic cardiomyopathies induced by chronic and binge ethanol (EtOH) feeding and characterize detailed hemodynamic alterations, mitochondrial function, and redox signaling in these models.	Literature review	54 studies	N/A	N/A	N/A	Chronic and binge EtOH feeding was characterized by contractile dysfunction, impaired relaxation, and vascular dysfunction. This was accompanied by enhanced myocardial oxidative/nitrative stress and deterioration of mitochondrial complex I, II, and IV activities and mitochondrial biogenesis, excessive cardiac steatosis, and higher mortality. Collectively, chronic plus binge EtOH feeding in mice leads to alcohol-induced cardiomyopathies characterized by increased myocardial oxidative/nitrative stress, impaired mitochondrial function and biogenesis, and enhanced cardiac steatosis.
Albakri A: *Alcoholic cardiomyopathy: A review of literature on clinical status and meta-analysis of diagnostic and clinical management methods *[11]	To combine current research findings to advance knowledge of alcoholic cardiomyopathy with a view of improving diagnosis and clinical management.	Literature review	128 studies	N/A	N/A	N/A	The prevalence of ACM varies significantly across regions ranging between 23% and 47% in DCM patients partaking excessive alcohol. The etiopathogenesis of ACM is not well understood, but it is hypothesized to be variable and inter-related pathologic mechanisms, mostly involving the generation of oxidative stress, myocyte apoptosis, impaired mitochondrial bioenergetics, and dysfunction in fatty acid metabolism and transport.
Steiner JL, Lang CH: *Etiology of alcoholic cardiomyopathy: Mitochondria, oxidative stress and apoptosis *[12]	To provide an unbiased review of the new and important discoveries related to the role and association between mitochondria, oxidative stress, and apoptosis in the development of ACM.	Literature review		Only studies published between 2010 and 2017 were included.	N/A	N/A	Alcohol impairs mitochondria function assessed by membrane potential and respiratory chain activity. Indictors of oxidative stress, including superoxide dismutase, glutathione metabolites, and malondialdehyde, are also adversely affected by alcohol oftentimes in a sex-dependent manner. In addition, myocardial apoptosis is increased based on assessment of terminal deoxynucleotidyl transferase dUTP nick end labeling (TUNEL) staining and caspase activity.
Nadruz W, Shah AM, Solomon SD: *Diastolic dysfunction and hypertension *[13]	To review the effects of hypertension on LVDD development through different mechanisms.	Literature review	64 studies	N/A	N/A	N/A	Blood pressure lowering using antihypertensive medications can improve LVDD; however, it remains unclear whether this improvement in LV diastolic function can improve cardiovascular outcomes.
Mirijello A, D’Angelo C, Ferrulli A, et al.: *Identification and management of alcohol withdrawal syndrome* [14]	To provide a practical tool for the identification and management of AWS, with a focus on pharmacotherapy	Literature review	121 Studies	N/A	N/A	N/A	Severe withdrawal could require ICU admission and the use of barbiturates or propofol. Other drugs, such as α2-agonists (clonidine and dexmetedomidine) and β-blockers, can be used as adjunctive treatments to control neuroautonomic hyperactivity. Furthermore, neuroleptic agents can help control hallucinations.
Maisch B: *Alcoholic cardiomyopathy *[15]	To review the effects of ethanol on the cardiovascular system, including aspects of inflammation [16], rhythm disturbances, and hypertension.	Literature review	131 studies	N/A	N/A	N/A	One to two drinks in men and one drink in women will benefit the cardiovascular system over time, one drink being 17.6 ml 100% alcohol. Moderate drinking can reduce the incidence and mortality of coronary artery disease, heart failure, diabetes, and ischemic and hemorrhagic stroke. More than this amount can lead to alcoholic cardiomyopathy, which is defined as alcohol toxicity to the heart muscle itself by ethanol and its metabolites.
Amor-Salamanca A, Guzzo-Merello G, González-López E, et al.: *Prognostic impact and predictors of ejection fraction recovery in patients with alcoholic cardiomyopathy *[16]	To define the role of LVEF improvement in the long-term outcome of ACM and to identify predictors of LVEF recovery in these patients.	Analysis of patients and the care outcomes	101 ACM patients	Only patients with AC were included in the study.	N/A		The LVEF recovery is associated with an excellent prognosis in ACM. Beta-blocker treatment, QRS <120 ms and absence of diuretics are independent predictors of LVEF recovery. LVEF recovery is similar in moderate drinkers and abstainers.
Shaaban A, Gangwani MK, Pendela VS, et al.: *Alcoholic cardiomyopathy *[17]	To review the evaluation of a patient with ACM, summarize the treatment options for ACM, describe the pathophysiology of ACM, and explain the importance of improving care coordination among interprofessional team members to improve outcomes for patients affected by ACM.	Literature review	18 studies	N/A	N/A	N/A	Long-term survival is directly associated with both the amount of alcohol use and the duration of this usage. Alcohol-induced dilated cardiomyopathy has a better prognosis than ischemia-induced cardiomyopathy. Atrial fibrillation, QRS widening of >120 ms, and absence of beta-blockers are associated with poor outcomes. Complications for those who continue to drink alcohol may include progressive heart failure, arrhythmias, and cardioembolic phenomenon. Data reveal that depending on the alcohol consumed, mortality rates of 40-80% are seen within 10 years.
O'Keefe JH, Bhatti SK, Bajwa A, DiNicolantonio JJ, Lavie CJ: *Alcohol and cardiovascular health: the dose makes the poison…or the remedy *[18]	To evaluate the effects of increased alcohol intake on cardiovascular health.	Literature review		N/A	N/A	N/A	The risk-to-benefit ratio of drinking appears higher in younger individuals, who also have higher rates of excessive or binge drinking and more frequently have adverse consequences of acute intoxication. Of the various drinking patterns, daily low- to moderate-dose alcohol intake, ideally red wine before or during the evening meal, is associated with the strongest reduction in adverse cardiovascular outcomes.
